# The Influence of Chestnut Extract and Its Components on Antibacterial Activity against *Staphylococcus aureus*

**DOI:** 10.3390/plants12102043

**Published:** 2023-05-20

**Authors:** Sara Štumpf, Gregor Hostnik, Tomaž Langerholc, Maša Pintarič, Zala Kolenc, Urban Bren

**Affiliations:** 1 Laboratory of Physical Chemistry and Chemical Thermodynamics, Faculty of Chemistry and Chemical Engineering, University of Maribor, Smetanova 17, 2000 Maribor, Slovenia; 2 Department of Microbiology, Biochemistry, Molecular Biology and Biotechnology, Faculty of Agriculture and Life Sciences, University of Maribor, Pivola 10, 2311 Hoče, Slovenia; 3 Department of Applied Natural Sciences, Faculty of Mathematics, Natural Sciences and Information Technologies, University of Primorska, Glagoljaška 8, 6000 Koper, Slovenia; 4 Institute of Environmental Protection and Sensors, Beloruska 7, 2000 Maribor, Slovenia

**Keywords:** tannins, antibacterial activity, MIC, MBC, *Staphylococcus aureus*, lag phase, generation time

## Abstract

Increasing antimicrobial resistance has caused a great interest in natural products as alternatives or potentiators of antibiotics. The objective of this study was to isolate individual tannins from crude chestnut extract as well as to determine the influence of both crude extracts (tannic acid extract, chestnut extract) and individual pure tannins (gallic acid, vescalin, vescalagin, castalin, castalagin) on the growth of Gram-positive *Staphylococcus aureus* bacteria. Their antibacterial activity was monitored by measuring the minimum inhibitory concentration (MIC) and minimum bactericidal concentration (MBC) as well as the duration of the lag phase, growth rate and generation time. The effect of growth medium strength on the MIC of different tannins was also investigated. Bacterial growth was followed spectrophotometrically, and MIC values were determined by the microdilution method. The MIC values of various isolated compounds allowed us to determine the bioactive compounds and their contribution to antimicrobial activity. It was found that MIC values increase with increasing growth medium strength and that the lag phase lengthens with increasing tannin concentrations, while the growth rates decrease. Comparing the results of the two studies, the antimicrobial activity of tannins against *S. aureus* was not as pronounced as in the case of *E. coli*, which may indicate that a different mechanism of action is responsible for the antimicrobial effects of tannins on Gram-positive than on Gram-negative bacteria, or that a different mechanism is more pronounced.

## 1. Introduction

Antimicrobial resistance represents a growing problem worldwide and poses a significant threat to human health. According to Murray et al. [1], an estimated 4.95 million deaths were associated with antimicrobial resistance in 2019, including 1.27 million deaths attributable to bacterial antimicrobial resistance. The increase in antibiotic resistance is due to several factors, including inappropriate antibiotic prescription and the use of antibiotics outside the healthcare sector. As a result, new antibacterial agents are needed to combat bacteria. Structural modifications of antimicrobials to which resistance has developed have already proven effective in expanding the use of antimicrobial agents [2]. Another way to combat bacteria involves the use of natural products, which can also act as alternatives or potentiators of antibiotics, since plants and different natural products contain an extremely large number of compounds of various origins with a wide range of bioactive properties, such as antioxidant, anticarcinogenic, anti-inflammatory, cardioprotective [3,4,5] and antibacterial, among others [6]. Plant-derived products represent around 25% of drugs in current clinical use [7,8,9].

The sweet chestnut (*Castanea sativa*) is a tree species from the foxtail family Fagaceae, native to Southern Europe and Anatolia and cultivated in parts of the world with temperate climates. Its wood is medium hard and heavy. It is durable in both dry conditions and water and is resistant to moisture fluctuations; therefore, it is used for making furniture, barrels, ships and carvings [5,10,11]. Chestnut is a good source of polyphenols, gallic acid and ellagic acid as well as of hydrolysable and condensed tannins [12]. Tannins represent natural bioactive compounds that are divided into two types, namely hydrolysable and condensed tannins [13,14]. Condensed tannins, also known as proanthocyanidins, are oligomers or polymers of flavan-3-ol linked by an interflavan carbon bond. On the other hand, hydrolysable tannins are esters of a polyol that is usually β-D-glucose with gallic acid (gallotannins) or ellagic acid (ellagitannins) [5,15,16]. Some plant species produce either hydrolysable or condensed tannins, whereas species like *Acacia*, *Acer*, *Castanea* and *Quercus* are known to contain both hydrolysable and condensed tannins. Whereas *Quercus* Sp. produces gallotannins and ellagitannins, *Castanea* produces only ellagitannins [17,18]. The latter are found in the fruit, stems, seeds, barks, wood and leaves of trees [19]. Different parts of the chestnut tree are used for tannin extraction, and these extracts are applied in the leather and food industries, as animal feed and in wine and spirit production [17,20,21]. 

The pathogens most commonly responsible for deaths due to antimicrobial resistance (AMR) include *Escherichia coli*, *Staphylococcus aureus*, *Klebsiella pneumoniae*, *Streptococcus pneumoniae*, *Acinetobacter baumannii*, and *Pseudomonas aeruginosa*. In 2019, these pathogens accounted for approximately 73.15% of AMR-attributable deaths and 72.12% of AMR-associated deaths [1]. The underlying mechanism of antimicrobial activity may differ between Gram-positive and Gram-negative bacteria, because the chemical composition of the cell walls of the two is different. The following mechanisms have been proposed for their antimicrobial activity: interaction of tannins with bacterial and substrate proteins [22,23,24], disruption of outer cell membrane integrity [5,25,26] and chelation of metal ions [22,27,28]. 

Many studies have established that tannin-rich extracts and the compounds contained in them exert antimicrobial activity [26,29,30,31]. However, it must be also emphasized that many studies on the antimicrobial activity of tannins have been performed on crude plant extracts and not on pure compounds, which makes it difficult to assess the contribution of each compound to the antimicrobial activity and to determine composition-to-activity and structure-to-activity ratios. In order to obtain this knowledge, studies on well-characterised materials as well as on purified compounds are required. The present study aims to provide a comprehensive insight into the effects of crude extracts and tannins isolated from chestnut against *Staphylococcus aureus*. We also wanted to gain additional insight into the mechanism of antibacterial activity of tannins against Gram-positive pathogenic bacteria; therefore, the effect of growth medium strength was investigated by changing the nutrient content available to the bacteria.

## 2. Results and Discussion

### 2.1. Identification of Isolated Vescalin, Castalin, Vescalagin and Castalagin

In order to understand which compounds contribute to the antimicrobial activity of chestnut extract, the individual compounds were first isolated from the crude extract. The description of the isolation procedure and the characterization of vescalagin and castalagin was provided in our previous study [6], and only the most important information is described here. The vescalagin and castalagin used in this study had chromatographic purities of 97.0% and 95.9%, respectively.

The structures of isolated vescalin and castalin were confirmed by a combination of methods. The HPLC retention time was compared to the retention time of the standard, and the chromatograms of castalin and vescalin are provided in Supplementary Material Figures S1 and S2, respectively. The structure of both compounds was further confirmed by LC–MS/MS and NMR spectroscopy techniques.

**Figure 1:**
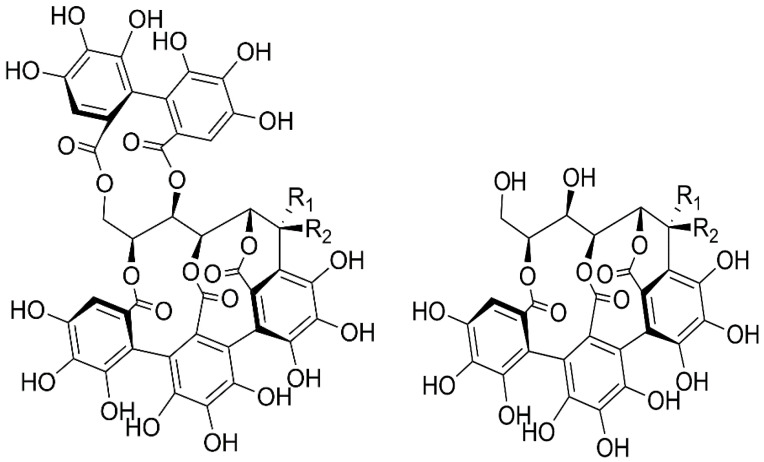
On the left are vescalagin (R1 = H, R2 = OH) and castalagin (R1 = OH, R2 = H), and on the right are vescalin (R1 = H, R2 = OH) and castalin (R1 = OH, R2 = H).

The chromatographic purities of isolated vescalin and castalin were 94.0% and 93.0%, respectively. Vescalin: ^1^H NMR (300 MHz, D2O) δ 6.90 (s, 1H); 5.43 (s, 1H); 5.13 (m, 1H); 4,86 (d, 1H, J = 1.68 Hz); 4.57 (d, 1H, J = 6.80 Hz); 4.06 (m, 1H); 3.94 (m, 2H). Castalin: ^1^H NMR [300 MHz, D2O] δ 6.90 (s, 1H); 5.70 (d, 1H, J = 4.10 Hz); 5.24 (d, 1H, J = 4 Hz); 5.14 (m, 1H); 5.00 (d, 1H, J = 6.70 Hz); 4.08 (t, 1H, J = 7.40 Hz); 3.94 (m, 2H). Structures of vescalin, castalin, vescalagin, and castalagin are depicted in Figure 1. 

### 2.2. Variation of the Growth Medium Strength and Its Influence on MIC

In this study, the influence of the bacterial growth medium strength on the minimum inhibitory concentration (MIC) of tannins against Gram-positive *S. aureus* was determined. This was performed in order to gain insight into the importance of the interactions of tannins with the growth medium for their antibacterial activity. The nutrient content in the growth medium varied from half to one and a half of the concentration recommended by the producer. 

The influence of growth medium strength on the MIC of tannins against Gram-negative bacteria *E. coli* was investigated previously [6], and a pronounced dependence of the MIC values on growth medium strength was observed. It was found that the rise in MIC values of all samples was approximately linear when the concentration of medium was increased, indicating that a direct interaction of tannins with the nutrients of the growth medium represents a likely source of their antimicrobial activity. Tannins have already been shown to chelate metal ions [32].

**Figure 2:**
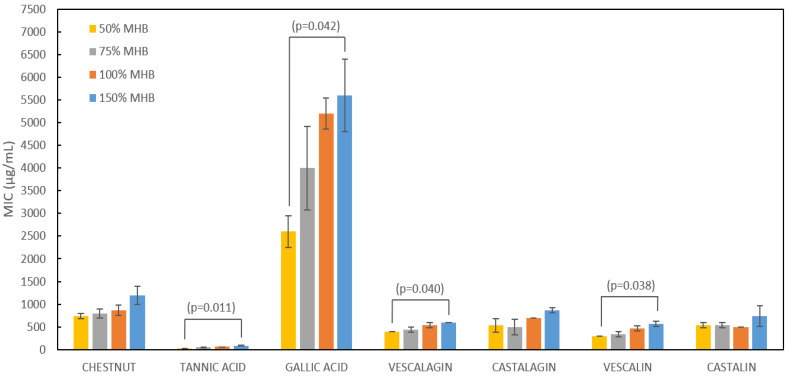
MIC values of the crude chestnut extract and its compounds in differently concentrated growth media. *p* values above brackets indicate the adjusted significance of pairwise comparison of MIC values in different medium strengths at the 0.05 significance level. All *p*-values are based on the Kruskal–Wallis test, and significance values were adjusted by Bonferroni correction. Error bars denote standard deviations of the mean.

The effect of the growth medium strength on Gram-positive *S. aureus* is depicted in Figure 2, while numerical values can be found in Supplementary Material Table S1. It can be further observed that with higher growth medium concentrations, the MIC values of almost all tested samples also increase. 

The Spearman’s rho correlation coefficient between the growth medium strength and MIC values is 0.28 and is at the 0.01 level of significance. Statistically significant differences were found between 50% growth medium concentration MIC and 150% growth medium concentration MIC for almost all samples except chestnut, castalagin and castalin. The increase in MIC values with increasing growth medium strength continued to be observed to some extent, but was not as pronounced as in the case of *E. coli* [6]. In the case of *E. coli*, the MIC values doubled with a doubling of the growth medium strength, indicating that the most pronounced mechanism of their antimicrobial action could be the complexation of tannins with essential ions in the growth medium. However, additional studies must be performed to prove this point. The only exception in the case of *S. aureus* were the MIC values of gallic acid, wherein the increase in MIC values was well pronounced. The dependence of the MIC value on the strength of the medium, although not as pronounced, was nevertheless present and indicates that the concentration of the medium has an influence on the strength of the compound, which in turn influences the MIC values. In the study by Araújo et al. [33], it was postulated that the most important mechanism of antimicrobial activity of vescalagin and castalagin against *S. aureus* is the disruption of the cell wall caused by the modulation of the normal arrangement of peptidoglycans on the bacterial surface. The main difference is that *S. aureus* belongs to the category of Gram-positive bacteria, which differ from Gram-negative bacteria (*E. coli*) in the chemical composition of the cell wall. Gram-positive bacteria possess a thicker layer of peptidoglycans, whereas Gram-negative bacteria possess a thinner layer of peptidoglycans and an additional outer membrane with lipopolysaccharides. The difference in cell wall structure between Gram-positive and Gram-negative bacteria may at least partly explain the different trends in the growth medium strength’s effect on tannins’ efficacy against *S. aureus* and *E. coli*. However, for additional confirmation, it would be beneficial to carry out a study on more representatives of Gram-positive bacteria.

### 2.3. Minimum Inhibitory Concentrations (MICs) of Studied Samples against Staphylococcus aureus

MIC values for all samples against *S. aureus* were determined visually using a broth microdilution assay. For comparison with the literature, the results of MIC values of tannins in 100% medium are collected in Table 1. The MIC values for media of different concentrations are summarised in Supplementary Material Table S1. 

All tested samples affected the growth of *S. aureus,* with MIC values ranging from 60 to 5200 µg/mL, whereas the MIC value of the positive control (streptomycin) was 3.25 µg/mL. It can be concluded that the MIC of gallic acid is an order of magnitude higher than that of all other studied samples, including the crude chestnut extract. However, the MIC of tannic acid was one order of magnitude lower than the other samples. The reason for the higher antibacterial activity of tannic acid may be found in the higher flexibility of the compounds, which makes interactions with proteins [34,35] or with the phospholipid bilayer [36] more likely. The MIC values of the crude chestnut extract and its isolated compounds are of the same order of magnitude. Castalagin exhibited a slightly stronger antibacterial activity than the chestnut extract itself (MIC value of 700 µg/mL of castalagin, compared to MIC value of 867 µg/mL for the chestnut extract). The MIC value of vescalagin was already slightly lower, with a value of 533 µg/mL. Finally, the MIC values of vescalin and castalin were practically half of the MIC value of the chestnut extract, with MIC values of 350 µg/mL and 450 µg/mL, respectively. It is interesting to observe that although vescalin and castalin, as well as vescalagin and castalagin, are structurally very similar compounds (they represent diastereoisomers), the difference in their stereochemistry obviously plays a role in their antibacterial activity, since the MIC values of vescalagin and vescalin were consistently lower than the MIC values of castalagin and castalin. When the MIC values were converted to molar rather than mass concentrations, the MIC values for vescalagin, castalagin, vescalin, and castalin were 0.57 µmol/mL, 0.75 µmol/mL, 0.55 µmol/mL and 0.71 µmol/mL, respectively. As already discussed above, the MIC values of vescalagin and vescalin were again consistently lower than the MIC values of castalagin and castalin. Furthermore, the MIC values of vescalagin and vescalin were now identical within the standard deviation. The same holds true for the MIC values of castalagin and castalin, possibly indicating that the common nonahydroxytriphenoyl group (NHTP group) is of crucial importance for the antibacterial activity of these four compounds.

The MIC values of some of the studied compounds for *S. aureus* have been already determined in previous studies. Araújo et al. [33] determined the MICs of vescalagin and castalagin against *S. aureus* (ATCC 25923) and methicillin-resistant *S. aureus* (ATCC 700698), and the results for *S. aureus* (ATCC 25923) are consistent with ours, with slight differences regarding the MIC values of vescalagin and those of castalagin. Our determined MIC value of vescalagin was 533 µg/mL, whereas theirs was 500 µg/mL, and our MIC of castalagin was 700 µg/mL, whereas their MIC was 500 µg/mL. Any discrepancy can be at least partly a consequence of the different dilution methods. Whereas most studies have typically used the twofold serial dilution method, the concentrations in our study were denser, which allowed a more accurate determination of MIC values, which cannot be observed with the logarithmic dilution method.

Taguri et al. [37] studied the MIC values of castalagin and tannic acid against the same strain of *S. aureus.* Their MIC value of 100 µg/mL for tannic acid was slightly higher than ours, which was 60 µg/mL. This difference in the MIC value of tannic acid could at least partly have arisen from a difference in tannic acid composition, since tannic acid in general is not a pure compound, but a mixture of various gallotannins. Their determined MIC value for castalagin was also 100 µg/mL, which is quite a bit lower than the value of 700 µg/mL that was determined in our study.

In a previous study [6], we determined the MIC values of pure tannins and extracts against Gram-negative *Escherichia coli,* and all exhibited lower MIC values compared to Gram-positive *S. aureus*. In many cases, the MIC of antibiotics against Gram-positive bacteria was lower than against Gram-negative bacteria, because the latter have an additional outer membrane. In our case, it was just the opposite, which may be due to different mechanisms of the antibacterial action of tannins against Gram-negative and Gram-positive bacteria [2].

Antibacterial activity of tannic and gallic acid on the growth of *S. aureus* ATCC 6538P was also monitored by Chung [38] by using the disk diffusion method. Gallic acid at a concentration of 5 mg/mL showed no antibacterial activity, whereas 5 mg/mL tannic acid inhibited the growth of many Gram-positive and Gram-negative bacteria, which corresponds with our findings, as the MIC value of tannic acid was very low, whereas gallic acid had a very high one.

It should be noted that the MIC values reported in the literature vary to a certain extent. However, the values given for vescalagin and castalagin are, at least quantitatively, consistent.

### 2.4. Minimum Bactericidal Concentrations (MBCs) of Studied Samples against S. aureus

MBC values were determined for all samples, and the values for 100% medium are collected in Table 2. All values were higher than the corresponding MIC values. It should be noted that MBC values were determined for all tested samples except for tannic acid. 

As in the case of MIC, the highest MBC value was determined for gallic acid, which was also an order of magnitude higher than that of the remaining samples. The MBC value of the crude chestnut extract was 2000 µg/mL, whereas the MBC values of vescalagin and castalagin were slightly lower, at 1500 µg/mL. The MBC values of vescalin and castalin were lower still, at 1000 µg/mL. It was observed that for tannic acid, whose MIC values were significantly lower than those of the remaining samples, the bacteria regrew after subculturing on the agar plates at all concentrations up to 500 µg/mL.

To the best of our knowledge, this is the first study determining the MBC of vescalin and castalin, whereas MBC values of vescalagin and castalagin against *S. aureus* (ATCC 25923) and methicillin-resistant *S. aureus* (ATCC 700698) were determined by Araújo et al. [33]. The reported values for *S. aureus* (ATCC 25923) were practically the same as the MBC values determined in our study (1000 µg/mL), whereas the reported values for strain ATCC 700698 were somewhat lower (250 µg/mL). 

### 2.5. Lag Phases, Growth Rates and Generation Times

**Figure 3:**
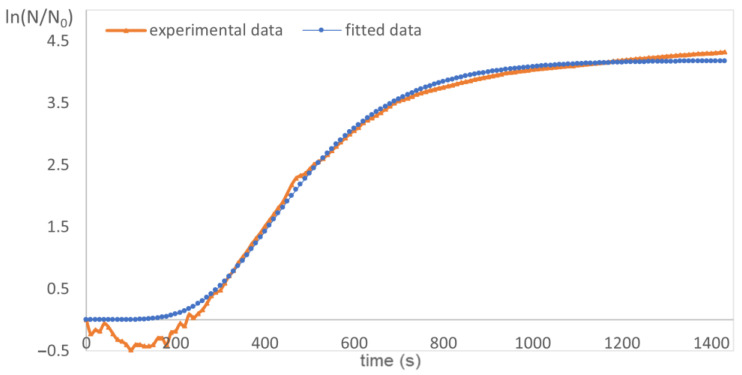
An example of experimental and fitted data, applied for the determination of the growth rate and lag phase duration.

Experimentally obtained growth curves were fitted to Equation (1), resulting in corresponding growth rates and lag phases [39]. The results of the fit agreed very well with the experimentally obtained data, as can be observed in Figure 3. Generation times were calculated from Equation (2). Both equations are detailed in Section 3.6.

As can be observed from Table 3, the lag time of the negative control was 100 min, which agrees well with lag times reported in the scientific literature, wherein the length of the lag phase was around 120 min [40]. The lag times of *S. aureus* lengthened with the increasing tannin concentration for the vast majority of samples. It can also be observed that all samples had a longer lag phase than the negative control even at the lowest tannin concentrations. The growth rates also decreased for the majority of samples. However, some concentrations deviated from these general trends. Since generation times were calculated directly from the growth rates, the generation times also deviated in these cases, but generally lengthened with the increasing tannin concentrations. This phenomenon is a consequence of environmental challenges or cellular stresses that may often prolong the lag phase and simultaneously cause low growth rates of a particular microbial population [41]. 

However, the observed extensions of the lag phases of *S. aureus* were not as pronounced as in the case of *E. coli* [42]. In the case of *E. coli*, the prolongation of the lag phase followed an exponential relationship with the increasing tannin concentration, which we assumed to be due to the chelation of certain essential ions from the growth medium with tannins. In the case of *S. aureus*, no exponential elongation was observed. This could be due to the fact that a different mechanism of tannin antimicrobial action was taking over. 

## 3. Materials and Methods

### 3.1. Antibacterial Agents

The materials tested as antimicrobial agents in this study were Streptomycin, tannic acid, chestnut extract and the pure compounds gallic acid, vescalin, castalin, vescalagin and castalagin. Gallic acid (Lot: BCBV2518), Streptomycin sulfate salt (Lot: SLBF8077V) and tannic acid (Lot: BCBT8361) were purchased from Sigma-Aldrich, St. Louis, MO, USA. The chestnut extract (Farmatan) was purchased from Tanin Sevnica, Sevnica, Slovenia. The remaining compounds were isolated from the chestnut extract. Four pure tannin compounds were isolated (vescalagin, castalagin, vescalin, castalin). Vescalagin and castalagin were isolated according to the procedure described in a previous article [6] and their chromatographic purities were 95.9% and 97.0%, respectively. 

### 3.2. Isolation of Vescalin and Castalin

Both vescalin and castalin were isolated from the chestnut extract, and their original contents were 1.0% and 1.6%, respectively [6]. 

The chestnut extract was dissolved in a mixture of methanol and water (50/50, *v*/*v*). The obtained solution was centrifuged and filtered. Methanol was removed using a rotary evaporator (Buchi, Essen, Germany). Several separation and purification steps were performed using a puriFlash^®^ 5.250 chromatography system (Interchim SA, Montlucon, France) equipped with autosampler, UV/Vis and ELSD detectors. Initially, two purifications were performed on the flash chromatographic column PF-50C18HP-F0004 with a stationary phase Sephadex LH-20. The solvents and gradient used are listed in Table 4. 

After each step, the fractions were analysed by the HPLC method described below, and all fractions rich in vescalin and castalin were pooled and freeze-dried. These two steps helped us to remove the majority of impurities and partially purify the compounds, but vescalin and castalin were still not separated.

Two flash purifications on a stationary phase Sephadex LH-20 were followed by purification on a flash reverse-phase (C18) chromatographic column PF-50C18HP-F0025. Samples were dissolved in type 1 water and filtered through a 0.2 μm PTFE syringe filter. In Table 5 are listed the solvents and gradient that were applied. 

As after separation with Sephadex LH-20, the fractions were analysed by HPLC, and three fractions were obtained, the first rich in vescalin, the second rich in castalin, and the third not separated. The organic solvents were removed using a rotary evaporator, whereas water was removed by lyophilization.

To completely separate and further purify the samples, three consecutive purifications were performed on the preparative reverse-phase (C18) chromatographic column US10C18HQ-250/212. The solvents and gradient applied were the same as for the flash reverse-phase chromatography. Fractions were analysed by HPLC, and sufficiently pure vescalin rich fractions or castalin rich fractions were joined and lyophilized using a freeze dryer.

### 3.3. Identification of Isolated Compounds Using HPLC

An analytical HPLC system (Vanquish Core, Thermoscientific, Waltham, MA, USA) was utilized to analyse the composition of each fraction after each purification and to determine the purity of the final isolates. The column used was an Agilent Eclipse XDB-C18 with 5 µm particles and dimensions of 4.6 × 150 mm. Two mobile phases were applied and are listed in Table 6, along with the gradient used.

### 3.4. Antibacterial Assay

The antimicrobial activity of the tested samples was determined by means of minimum inhibitory concentration (MIC) using the broth microdilution assay previously described, with slight modifications [6], and MBC. 

To determine the effect of growth media on the MIC, the nutrient concentration was varied by changing the concentration of MHB to from half to one and a half the concentration recommended by the manufacturer. 

#### 3.4.1. Microorganisms and Culture Conditions

The strain used in this study was Gram-positive *S. aureus* ATTC 29213. Bacteria were cultured in Mueller-Hinton broth (MHB; Sigma-Aldrich) and incubated at 37 °C for 24 h. The final density of the culture was 2–3 × 10^5^ CFU/mL, which was confirmed by regular colony counts during the study. A fresh overnight culture was established for each experiment.

#### 3.4.2. Preparation of Antimicrobial Agents

Prior to the analysis, all samples were dissolved in DMSO (DMSO, Sigma-Aldrich), since they are not sufficiently soluble in MHB. Samples were serially diluted with MHB to obtain densely decreasing concentrations. The maximum final concentration of DMSO did not exceed 5% *v*/*v*. 

#### 3.4.3. Broth Microdilution Assay

To determine the influence of the growth medium strength on the MIC, different concentrations of Mueller–Hinton broth (MHB), the bacterial growth medium, were prepared. The concentrations of the media were 50%, 75%, 100% and 150% of the concentration recommended by the producer.

Bacteria were incubated with antimicrobial agents in different concentrations of the MHB at 37 °C. All tests were performed in triplicates and results were calculated as means ± standard deviation.

#### 3.4.4. Determination of Minimum Inhibitory Concentration (MIC)

MIC was determined visually, and MIC values were reported as means. MIC (µg/mL) represents the lowest concentration of an antimicrobial agent that prevents the growth of microorganisms. 

#### 3.4.5. Determination of Minimum Bactericidal Concentration (MBC)

Minimum bactericidal concentrations (MBC) were measured by subculturing 100 µL samples from microplates incubated for 24 h. Samples from wells with tannin concentrations determined to be MIC and higher were subcultured onto MH agar plates. The agar plates were incubated at 37 °C for 24 h. MBC values were determined as the lowest concentrations of a sample in which ≥99.9% of the original bacterial inoculum was killed.

### 3.5. Statistical Analysis

The correlation between medium strength and MIC values was determined using SPSS Statistic. Spearman’s rho correlation coefficient was applied, as well as the Kruskal–Wallis test. Finally, significance values were adjusted using the Bonferroni correction. 

### 3.6. Lag Phases, Growth Rates and Generation Times

Bacteria were incubated with antimicrobial agents at 37 °C (±0.5 °C) and measured every 10 min for 24 h at 595 nm using a spectrophotometer: Tecan Infinite 1000PRO. Growth curves were determined by plotting the time dependence of optical density (OD). Then, the experimentally obtained growth curves were fitted to Equation (1) analogously to the article by Li et al. [39]. In this way, the bacterial growth parameters (growth rates and lag phases) were determined, and the results of the fit agreed very well with the experimentally obtained data.

(1) $$\ln\frac{N}{N_{0}}=A\exp(-\exp(\frac{\mu e}{A}\left(\lambda -t\right)+1))$$

In Equation (1), *N* represents the cell number at time *t*, *N*_0_ represents the cell number at the beginning of the experiment, *µ* represents the maximum growth rate at the exponential phase, *λ* represents the lag phase duration and *A* represents the maximum OD throughout the experiment. Generation times were obtained from Equation (2),

(2) $$t_{d}=\frac{\ln\left(2\right)}{\mu }$$

where μ represents the maximum growth rate during the exponential phase. 

## 4. Conclusions

The antibacterial activity of a crude chestnut extract, as well as of its five main compounds (vescalagin, castalagin, vescalin, castalin, and gallic acid) and tannic acid against *S. aureus* were determined using minimum inhibitory concentration (MIC) and minimum bactericidal concentration (MBC). All investigated samples tested were found to have antimicrobial activity against *S. aureus*, with tannic acid and gallic acid exhibiting the lowest and the highest MIC values, respectively. The MIC values of vescalagin and castalagin are in good agreement with the ones reported in the scientific literature. It was interesting to observe that the reported MICs in molar concentrations were virtually the same for vescalin and vescalagin as well as for castalin and castalagin, which indicates that the nonahydroxytriphenoyl group (NHTP group) plays an important role in the antibacterial activity of the studied tannins. Moreover, the MIC values of both vescalin and vescalagin were found to be systematically lower than the MIC values of their corresponding diastereoisomers castalin and castalagin. As the concentration of the investigated tannins increased, the lag phases lengthened, whereas the growth rates decreased. Finally, the effect of the growth medium strength was also observed by varying the nutrient content of the medium. A stronger growth medium led to an increase in MIC values. However, this effect was not as pronounced as in the case of *E. coli*, studied in the previous work, wherein it was observed that essential ion complexation with tannins might affect MIC values, which indicates that this is not the case for the antimicrobial activity of investigated tannins against the Gram-positive *S. aureus*. The observed differences between the influence of the growth medium strength on the growth of *E. coli* and *S. aureus* may also be due to the different structure of the cell walls of Gram-positive and Gram-negative bacteria. Further studies are needed to generalize these results for all Gram-positive and Gram-negative bacteria.

## Figures and Tables

**Table 1:** MIC values of chestnut extract and tannin compounds against *S. aureus* in 100% MHB. Reported errors are standard deviations from average MIC values.

Minimum Inhibitory Concentration (MIC)		
Sample	(µg/mL)	µmol/mL
chestnut extract	867 ± 115	/
tannic acid	60 ± 0	0.04 ± 0.00
gallic acid	5200 ± 346	27.64 ± 0.61
vescalagin	533 ± 58	0.57 ± 0.06
castalagin	700 ± 0	0.75 ± 0.00
vescalin	350 ± 58	0.55 ± 0.16
castalin	450 ± 0	0.71 ± 0.00
streptomycin	3.25 ± 0.87	0.004 ± 0.001

**Table 2:** Minimum bactericidal concentrations (MBCs) of chestnut extract and tannin compounds against *S. aureus*.

Minimum Bactericidal Concentration (MBC)	
Samples	(µg/mL)
chestnut extract	2000
tannic acid	^a^NI
gallic acid	10,000
vescalagin	1500
castalagin	1500
vescalin	1000
castalin	1000
streptomycin	8

^a^NI = No inhibition observed in concentrations up to 500 µg/mL.

**Table 3:** Growth rates (µ), lag times (lag) and generation times (t_d_) of investigated samples (in 100% media).

Samples	Concentration (µg/mL)	µ (min^−1^)	lag (min)	t_d_ (min)
negative control	0	0.0208	100	33
chestnut extract	400	0.0141	269	49
	500	0.0144	267	48
	600	0.0119	284	58
	700	0.0119	587	58
tannic acid	300	0.0109	189	64
	400	0.0074	299	94
	500	0.0101	289	69
gallic acid	2400	0.0141	179	49
	3000	0.0168	273	41
	3600	0.0120	312	58
	4200	0.0082	355	84
vescalagin	500	0.0126	513	55
castalagin	300	0.0113	247	61
	400	0.0098	253	71
	500	0.0085	260	81
	600	0.0056	407	123
vescalin	100	0.0168	281	41
	200	0.0144	382	48
	300	0.0111	537	62
castalin	400	0.0151	438	46
streptomycin	0.25	0.018	164	39
	0.5	0.012	220	57
	1	0.013	320	53
	2	0.0045	922	152

**Table 4:** The gradient, applied in two initial flash purifications. The solvents used were A: water with 0.1% formic acid; B: methanol; C: acetone.

t (min)	Solvent A (%)	Solvent B (%)	Solvent C (%)
0.0–60.0	100	0	0
60.4–135.0	70	30	0
135.4–185.0	50	50	0
185.4–197.5	90	0	10
197.9–210.0	70	0	30
210.4–222.5	50	0	50
222.9–240.9	25	0	75
248.3–260.0	50	50	0
260.4–309.1	100	0	0

**Table 5:** The gradient, applied in additional flash and preparative purifications. The solvents used were A: water containing 0.1% formic acid; B: acetonitrile.

t (min)	Solvent A (%)	Solvent B (%)
0.0–6.4	98	2
8.6–20.1	2	98
22.2–29.1	98	2

**Table 6:** The gradient applied in an analytical HPLC method. The solvents used were A: 0.1% solution of formic acid in water; B: acetonitrile.

t (min)	Solvent A (%)	Solvent B (%)
0.0—2.0	98	2
14.0	50	50
15.0–20.0	10	90
22.0–26.0	98	2

## Data Availability

Not applicable.

## Supplementary Materials

The following supporting information can be downloaded at: https://www.mdpi.com/article/10.3390/plants12102043/s1, Figure S1: HPLC chromatogram of castalin at 280 nm, Figure S2: HPLC chromatogram of vescalin at 280 nm, Table S1: Minimum inhibitory concentration (MIC) values of chestnut extract and its compounds in differently concentrated growth media.

## Abbreviations

AMR—antimicrobial resistance; MBC—minimum bactericidal concentration; MIC—minimum inhibitory concentration; NHTP group—nonahydroxytriphenoyl group; OD—optical density.
