# Supplementary material for: The Influence of Chestnut Extract and Its Components on Antibacterial Activity against Staphylococcus aureus
Source: Plants (Basel). 2023 May 20;12(10):2043. doi: 10.3390/plants12102043 (PMC10221632; doi:10.3390/plants12102043)
Supplement: Supplementary file 1 [file plants-12-02043-s001.zip › plants-2386125-supplementary.pdf]

Plants

Supplementary Material

The Influence of Chestnut Extract and Its Components on Antibacterial Activity of *Staphylococcus aureus*

Sara Štumpf [1], Gregor Hostnik [1], Tomaž Langerholc [2], Maša Pintarič [2], Zala Kolenc [1,2,3] and Urban Bren [1,3,4,*]

1 Laboratory of Physical Chemistry and Chemical Thermodynamics, Faculty of Chemistry and Chemical Engineering, University of Maribor, Smetanova 17, 2000 Maribor, Slovenia
2 Department of Microbiology, Biochemistry, Molecular Biology and Biotechnology, Faculty of Agriculture and Life Sciences, University of Maribor, Pivola 10, 2311 Hoče, Slovenia
3 Department of Applied Natural Sciences, Faculty of Mathematics, Natural Sciences and Information Technologies, University of Primorska, Glagoljaška 8, 6000 Koper, Slovenia
4 Institute of Environmental Protection and Sensors, Beloruska 7, 2000 Maribor, Slovenia

* Correspondence: urban.bren@um.si, Tel.: +386-2-2294-421

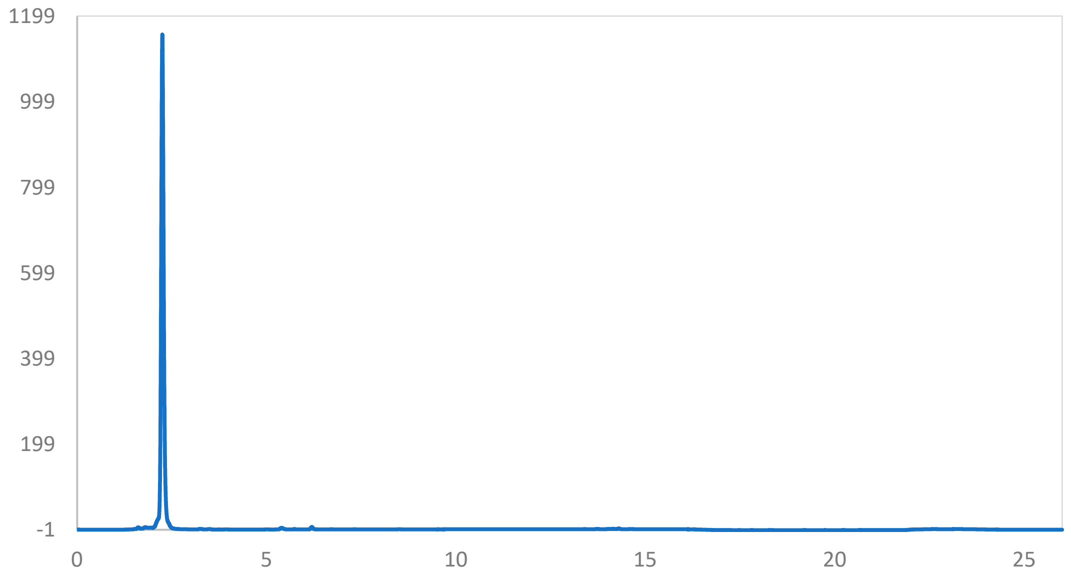

Figure S1. HPLC chromatogram of castalin at 280 nm.

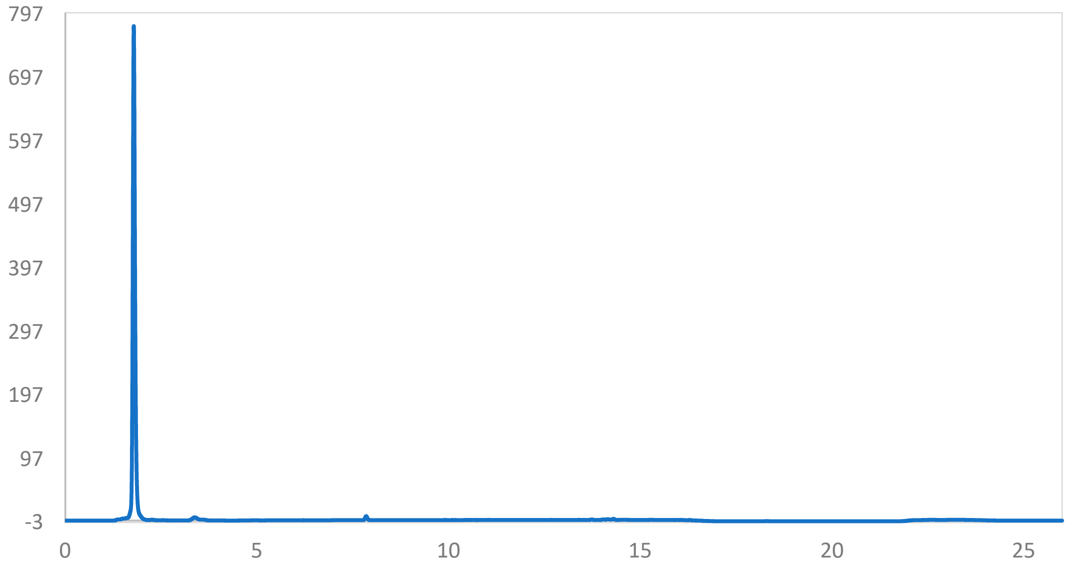

Figure S2. HPLC chromatogram of vescalin at 280 nm.

Table S1. Minimum inhibitory concentration (MIC) values of chestnut extract and its compounds in differently concentrated growth media.

	0.5	0.75	1	1.5
	MIC values in μg/mL			
Chestnut extract	733.33 ± 57.74	800.00 ± 100.00	866.67 ± 115.47	1200.00 ± 120.00
Tannic acid	23.33 ± 5.77	50.00 ± 10.00	60.00 ± 0.00	85.00 ± 8.66
Gallic acid	2600.00 ± 346.41	4000.00 ± 916.52	5200.00 ± 346.41	5600.00 ± 800.00
Vescalagin	400.00 ± 0.00	433.00 ± 57.74	533.33 ± 57.74	600.00 ± 0.00
Castalagin	533.00 ± 152.75	500.00 ± 173.21	700.00 ± 0.00	866.67 ± 57.74
Vescalin	300 ± 0.00	333.33 ± 57.74	350.00 ± 57.74	566.67 ± 57.74
Castalin	533.33 ± 57.74	533.00 ± 57.74	450.00 ± 0.00	733.33 ± 230.94

0.5 = half the concentration of *MHB* growth medium recommended by the producer; 0.75 = 75% of the concentration of *MHB* growth medium recommended by the producer; 1 = the concentration of *MHB* growth medium recommended by the producer; 1.5 = one and a half the concentration of *MHB* growth medium recommended by the producer.
